# Quantum confinement dominates band gaps while defects lead the photoluminescence in silicon nanowires

**DOI:** 10.1039/d6ra01273f

**Published:** 2026-05-18

**Authors:** Aarti Diwan, Tharun J, Chandeep EC, Anand Mohan Shrivastav, Tulika Srivastava, Rajesh Kumar, Shailendra K. Saxena

**Affiliations:** a Optics and Nanoelectronics Laboratory (ONE), Department of Physics and Nanotechnology, College of Engineering and Technology, SRM Institute of Science and Technology Kattankulathur Tamil Nadu 603203 India shailens@srmist.edu.in; b Department of Electronics & Communication, College of Engineering and Technology, SRM Institute of Science and Technology Kattankulathur Chennai 603203 India tulikas@srmist.edu.in; c Materials and Device Laboratory, Department of Physics, Indian Institute of Technology Simrol Indore India 453552

## Abstract

The quantum confinement effect has been observed in silicon nanowires (SiNWs) and is a widely accepted mechanism for visible photoluminescence (PL). Besides the observed widening of the band gap, an increase in Urbach energy is seen, revealing the presence of structural disorder due to oxygen-related defects. Surprisingly, in contrast to band gap widening, PL spectra showed a spectral shift instead of peak widening. Our finding indicates that oxygen-related defect states have a significant influence on the shifts in PL peaks. The combined influence of the quantum confinement effect and these defect states is demonstrated to play an important role in defining the PL response. This work provides insights into the interplay between quantum confinement and defect states, offering a clearer understanding of PL mechanisms in SiNWs.

## Abstract 2

Confinement effect results in band gap increase while defects result in nearly constant photoluminescence in SiNWs.

## Graphical abstract

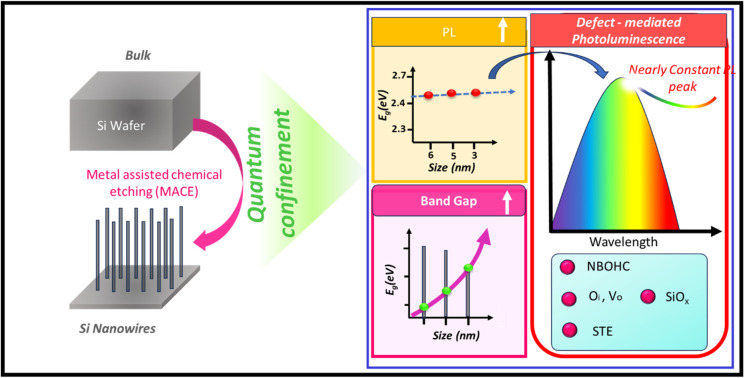

## 1. Introduction

The discovery of nanomaterials not only gave birth to a new era of technology but also led to the investigation of subtle physical phenomena happening at the nanoscale.^1,2^ Silicon, as one of the most studied semiconductors, offers a comparatively easy nanomaterial system to study nanoscale phenomena. Silicon nanowires (SiNWs) are highly attractive materials in semiconductor technology due to their growing appeal that stems from their unique characteristics such as visible light emission under ambient conditions, high-density electronic states, and surface reactivity.^3^ SiNWs are being considered as potential candidates for applications in sensing, photovoltaics,^4^ photodetection,^5^ light-emitting diodes (LEDs),^6^ and photocatalysis.^7^ Additionally, their high surface-to-volume ratio amplifies the influence of surface states and structural defects in introducing mid-gap energy levels capable of facilitating radiative transitions. Unlike bulk silicon, where defects often degrade performance, defects in SiNWs can be strategically engineered to enhance luminescence, opening new avenues for optoelectronic applications. Defects in silicon nanowires can arise during their synthesis process itself. Common growth techniques, such as the vapor–liquid–solid (VLS) method^8^ or metal-assisted chemical etching (MACE),^9,10^ often introduce structural irregularities. Previous studies have employed advanced nanowire growth techniques in conjunction with detailed spectroscopic investigations to systematically examine both the growth mechanisms and optical properties of semiconductor nanowires, thereby providing comprehensive insights into their confined electronic and optical characteristics.^11–13^

Visible photoluminescence (PL) at room temperature (RT) has been observed in several kinds of silicon nanostructures (SiNSs),^14^ including Si nanowires (SiNWs),^15^ although the mechanism is unclear. The proposed explanations for PL in SiNSs include (i) the presence of a native oxide layer including non-bridging oxygen hole centres (NBOHCs) and other oxygen-related defects,^14^ (ii) quantum confinement effects (QCE) alone or in combination with surface states,^16^ and (iii) direct band gap emissions in aligned SiNWs.^17^ A physical understanding of the formation mechanisms of defect-related luminescence is becoming increasingly important. Therefore, in this work, we analyse the discrepancy between the band gap energy and photoluminescence peaks of SiNWs. Here, we demonstrate the utilization of diffuse reflectance spectroscopy (DRS) and Urbach energy to understand the defect level identification in SiNWs. We focus on understanding the origin of strong visible PL from SiNWs. The dependence of the PL spectra of SiNWs on the size of SiNWs, etching parameters, and SiO_*x*_ is studied systematically. It is found that band gaps follow the quantum confinement effect, meaning the band gap increases with a decrease in the size of the nanostructure while PL does not, and PL arises at (almost) the same energy level originating from defect levels with the band gaps.

## 2. Experimental

An n-type Si wafer (100) with a resistivity of (0.01–0.2) Ω cm was used to make silicon nanowires (SiNWs) *via* the metal-assisted chemical etching (MACE) technique.^10,18^ Prior to the porosification process, the Si wafer was broken into three pieces and sequentially rinsed in ethanol, acetone, and isopropyl alcohol (IPA) for 10 min through ultrasonication.^19^ The cleaned wafers were immersed in a 5% HF solution to remove the native oxide layer. After that, Ag nanoparticles (AgNPs) were deposited by placing the wafers in a mixture of 5% HF and 5 mM AgNO_3_ for 60 seconds at room temperature.^2,14^ In the next step, the wafers were rinsed with deionized water to get rid of the extra Ag ions. Then, the Ag-decorated Si wafers were etched in an HF/H_2_O_2_ solution for 30 (S1), 45 (S2), and 60 (S3) minutes. After etching, the wafers were placed in HNO_3_ to dissolve AgNPs. Finally, the samples were put back in HF to remove any oxide layer that may have formed during the treatment with HNO_3_.^20,21^ The surface morphology of the SiNW samples was characterized by scanning electron microscopy (SEM) (Thermo Scientific Apreo S). Elemental composition analysis was performed using an energy dispersive X-ray spectrometer (EDX) attached to a FESEM system. Further, Raman spectroscopy was performed on a Jobin-Yvon Horiba micro-Raman spectrometer to identify the size of SiNWs. The optical properties of the materials were systematically studied by diffuse reflectance spectroscopy (DRS) using an Agilent UV-vis spectrometer (model Cary 60), and room temperature photoluminescence (RTPL) studies were carried out using a Dong Woo Optron 80K PL system.

## 3. Results and discussion

**Fig. 1:**
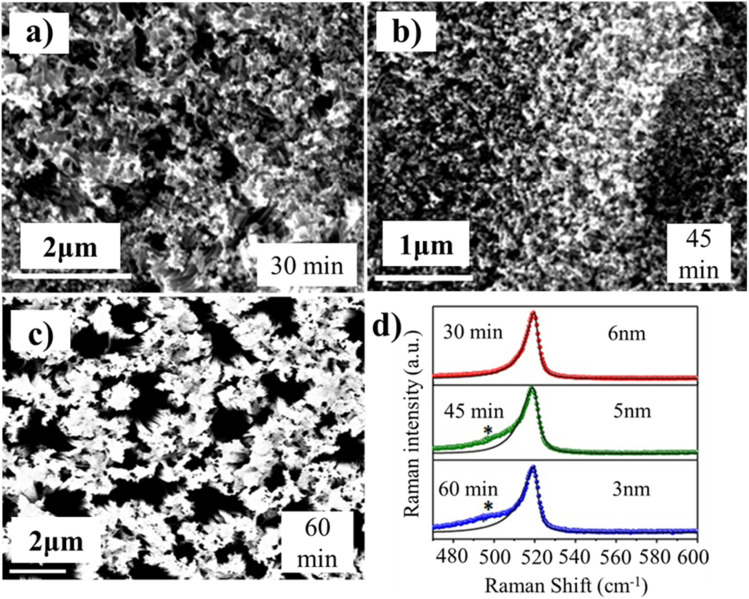
Surface morphologies of the etched Si surface at different etching times (a) 30 min, (b) 45 min, and (c) 60 min. (d) Raman spectra of SiNSs for the etching times of 30, 45, and 60 min.

The surface morphology of SiNWs was studied using scanning electron microscopy (SEM) at different etching times, as shown in Fig. 1a–c, revealing the mesoporous structure of SiNWs. The cross-sectional view of the SiNW samples is shown in the SI. These SiNWs are porous and contain nanostructures (NSs) in them.^22^ In order to investigate the nature and crystallinity, spectroscopy studies were performed.

**Fig. 2:**
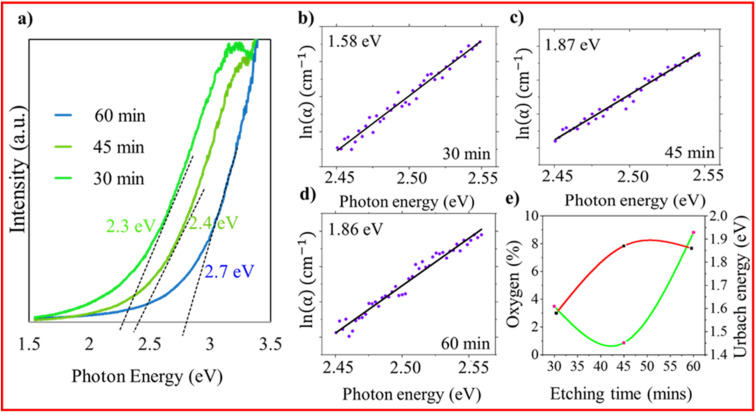
(a) Diffuse reflectance spectra of samples etched for 30, 45, and 60 min. (b) Urbach energy plot for 30 min (1.58 eV), (c) 45 min (1.87 eV) and (d) 60 min (1.86 eV). (e) Plot depicting the relation between oxygen (%), etching time (min), and Urbach energy.

Raman spectroscopy, a non-destructive yet fast technique,^23,24^ is utilized here to investigate the band gap of the SiNW samples, and the Raman spectra of all the samples are shown in Fig. 1d. The Raman spectra are red-shifted and asymmetrically broadened when compared to bulk Si (black solid lines), showing the confinement effect.^15,25,26^ Reports confirm that the shift in Raman spectra is caused by either the presence of individual silicon nanocrystals (SiNCs) within the nanowires (NWs) or the strain due to amorphous SiO_*x*_.^27–30^ The increasing prominence of defect-related features (marked with *), particularly between 45 and 60 min further reflects the enhanced structural disorder that accompanies progressive etching.^31^ The phonon confinement model (PCM) states that eqn (1) can be used to express the Raman line shapes to estimate the size of the nanostructure (NS) in the samples. The following equation was used to fit the experimental Raman data.1
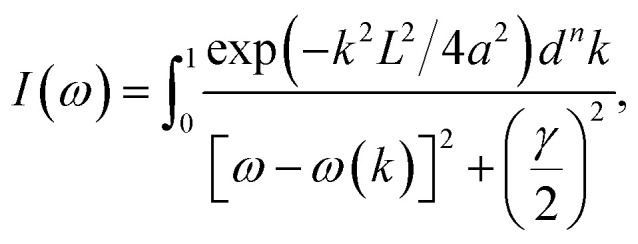
where *k* is reduced wave vector, *a* is the lattice parameter of the material (0.543 nm), *L* denotes the size of the NS present in the sample, *ω*(*k*) represents the phonon dispersion relation of the optical phonon in Si and is expressed as *ω*(*k*) = [*A* + *B* cos(π*k*/2)]^1/2^, where *A* = 171 400 cm^−2^ and *B* = 100 000 cm^−2^, *γ* is the FWHM of the Raman spectrum of the bulk material, and *n* is the order of confinement.^24^ As the etching duration increases from 30 to 60 min, the size of the NSs in the SiNW samples decreases as follows: 30 min (6 nm) > 45 min (5 nm) > 60 min (3 nm) (Fig. 1d). The estimated sizes by Raman line morphologies are comparable to Si's Bohr exciton radius, meaning that SiNW samples exhibit quantum confinement (QC).^25,32^ The confinement inferred from Raman analysis is corroborated with the optical responses obtained from DRS. The Kubelka–Munk method is used to verify QC in SiNWs. Fig. 2a shows the Tauc plot (using DRS data) of the samples etched for 30, 45 and 60 min, which reveals that the band gap of SiNWs increases with etching time, from 2.3 eV (30 min) to 2.4 eV (45 min) and 2.7 eV (60 min), indicating the QC effect.^20^ This effect implies that the band gap increases when the size of silicon nanostructures (SiNSs) decreases. These findings are consistent with the findings from Raman experiments: with higher etching time, the size of SiNSs decreases and band gap increases. To analyse the influence of defects on NWs, an Urbach tail estimation is carried out. We plotted Urbach energy, as a plot of ln(*α*) *versus* photon energy for each of the three samples (Fig. 2b–d), and a linear fitting was done on the linear part of the curve. Interestingly, Urbach energy increases from 1.58 eV to 1.87 eV as the etching time increases, attributed to the increase in band gap. It is widely noticed that when exposed to oxygen, Si tends to produce SiO_*x*_ and other oxygen-related defects, which can induce deformation on the lattice surface.^15,33^

For a better understanding, Fig. 2e illustrates the relationship between the oxygen (O) content (%), etching time, and Urbach energy. The O content (%) was measured by energy dispersive X-ray (EDX) analysis. As the etching time increases (red line), the percentage of oxygen and Urbach energy also increase (green line). It is important to note that for the etching time of 45 min, the percentage of O content is very low and Urbach energy is very high, possibly due to more oxygen vacancies (V_o_). V_o_ create localized electronic states and trap excitons; these energy states contribute to electronic disorder. STEs can induce local lattice distortions due to their interaction with the crystal lattice (lattice vibration phonon), enhancing the electron-phonon coupling.^34^ The existence of STEs and their resulting lattice distortions can lead to a broader and more pronounced Urbach edge, meaning a larger Urbach energy value.^35,36^ Notably, Urbach energy is mostly equal for all samples, but the percentage of oxygen is higher in the case of the 60 min-etched sample than in the 45 min-etched sample because of the presence of more oxygen-related defect states associated with the SiO_*x*_/Si interface in SiNWs. The etching process becomes irregular if the etching time or oxygen concentration is excessive. The silicon at the metal-silicon interface gets etched more rapidly than the silicon distant from the interface, resulting in imperfections or oxidation of silicon to produce the SiO_*x*_/Si interface, which happens easily on the surface of SiNWs.^37,38^ It is also important to mention here that the Urbach energies obtained for these samples are higher than those generally observed. It might be due to the combined effect of the defects related to the oxygen vacancies and the presence of amorphous Si (observed in the Raman data, Fig. 1d). A table showing higher Urbach energies is given in the SI.

**Fig. 3:**
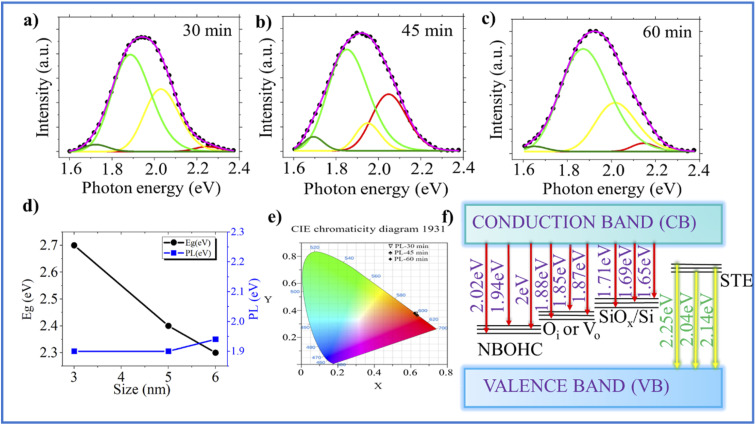
Deconvoluted PL spectra of the SiNW array at different etching times: (a) 30 min, (b) 45 min, and (c) 60 min. (d) Plot of band gap energy *versus* PL peak positions for varying nanostructure sizes, (e) CIE chromaticity diagram. (f) Band diagram of defect-related emissions.

Furthermore, room temperature photoluminescence (RTPL) measurements are essential for determining whether the emission originates from band–edge transitions or from defect-mediated processes.^39^ Thus, RTPL measurements were performed (Fig. 3a–c). Band gap energy and PL peak positions were analyzed for nanostructures of varying sizes. The band gap increases with a decreasing nanostructure size due to the quantum confinement effect, while the PL peak position remains nearly constant, indicating defect-related emissions. Different etching times exhibit clear variations in their emission characteristics in contrast to the monotonic increase in band gap energy obtained from diffuse reflectance spectroscopy (DRS) results (Fig. 3d). This discrepancy suggests that the observed photoluminescence does not originate from direct band-to-band recombination but rather from localized defect states.

We note that the deconvolution of the PL spectra at the etching durations of 30 min, 45 min, and 60 min exhibited four well-split peaks, indicating defect-related peaks. The findings are further validated by plotting the chromaticity coordinates of the PL data (on the 1931 standard), revealing the values of (0.63, 0.38), (0.62, 0.36), and (0.62, 0.35) for 30 min, 45 min, and 60 min, respectively (Fig. 3e). These coordinates confirm the presence of solely red emissions, with no evidence of green or blue emissions. These findings oppose the increase in band gap energy because PL corresponds to defect-related emissions. According to earlier reports, multiple defect states can exist within the SiNW lattice, including non-bridging oxygen hole centres (NBOHCs),^40,41^ excess interstitial oxygen (O_i_),^39^ oxygen vacancies (V_o_),^42,43^ self-trapped excitons (STEs),^44,45^ and SiO_*x*_/Si surface states.^38,46–48^ These defects are widely recognized as potential origins of defect-mediated PL in SiNSs.^40,49^ Therefore, transitions occur both from CB to NBOHC, O_i_, and V_o_ defect levels and from STE to VB, enabling multiple color emissions, as depicted in the band diagram (Fig. 3f). These include red emissions ((CB → NBOHC): Δ*E* ∼ (1.94 to 2 eV), (CB → O_i_ or V_o_): Δ*E* ∼ (1.85 to 1.87 eV), and (CB → SiO_*x*_/Si): Δ*E* ∼ (1.65 to 1.71 eV)) along with a yellow emission ((STE → VB): Δ*E* ∼ (2.04 to 2.25 eV)). Upon closely examining the PL spectra of the 30 min-etched sample, the deconvoluted peaks are at 1.71 eV, 1.87 eV, 2.02 eV, and 2.25 eV, but upon increasing the etching time, some significant changes are found in the PL peak positions. In the PL spectra of the 45 min-etched sample, the peak is shifted to the lower energy side between 1.85 and 2.04 eV due to the presence of NBOHCs, STEs, and higher V_o_ because the O content (%) is very less,^42^ which can be seen clearly (Fig. 2e). These V_o_ act as trapping sites and can increase the number of STE by creating new states within the band gap, which is responsible for higher Urbach energy^50,51^ (Fig. 2c). In the PL of the 60 min-etched sample, the peak from 1.87 to 2.14 eV is due to the excess O content or O_i_ as oxygen (%) increases sharply at 60 min (Fig. 2e).^41^ Similar to V_o_, NBOHCs can trap excitons.^52^ In the PL spectra of the 45 min-etched sample, this peak shifts to a slightly higher energy; it has been reported previously that in some cases, oxygen-rich Si also acts as a passivation layer for the trap sites, filling oxygen vacancies and increasing the optical gap due to the confinement effect.^53,54^ On the other hand, it is worth noting that the PL peak appears at 1.69 eV for the 45 min-etched sample, shifting to a lower energy at 1.65 eV for the 60 min-etched sample due to enhanced etching time, which is likely due to the presence of amorphous SiO_*x*_ on the surface; this is observed as peak broadening in the Raman spectra (Fig. 1d) when the etching time is more than 45 min.^46^ As the size of SiNWs decreases, surface oxidation increases, forming more SiO_*x*_/Si states, which lowers the luminescence.^31,33,55^ By correlating the PL and Raman studies, the key finding is a red shift observed in both spectra, attributed to the excessive oxygen vacancies and formation of SiO_*x*_, directly indicating increased Urbach energy.^31,43,56^ Therefore, this ambiguity in PL and band gap can be addressed by understanding the defect-related phenomenon.

## 4. Conclusion

An ambiguity in is observed the band gap and photoluminescence of SiNWs. The band gap from DRS is 2.3 to 2.7 eV at different etching times, indicating a substantial QC effect, which is aligned with our findings from Raman spectroscopy. The rise in Urbach energy relative to the band gap with prolonged etching suggests increased structural disorder and defect formation. Meanwhile, the PL spectra were deconvoluted, and it was found that the emission spectrum included red and yellow emission bands, indicating multiple defects in our samples. We explain that the PL spectra do not consistently indicate a corresponding higher energy shift in emission. In a few cases, a lower energy shift is observed. These apparent variations arise because PL is predominantly defined by the oxygen-related defects rather than changes in the band gap. This study addresses an important contradiction between the band gap and PL of SiNWs, providing a deeper understanding of the optical properties of SiNWs.

## Conflicts of interest

There are no conflicts to declare.

## Supplementary Material

RA-016-D6RA01273F-s001

## Data Availability

We have provided all the details in the supplementary information (SI). If any other relevant data are required, they can be provided on request. Supplementary information is available. See DOI: https://doi.org/10.1039/d6ra01273f.
