# Supplementary material for: Quantum confinement dominates band gaps while defects lead the photoluminescence in silicon nanowires
Source: RSC Adv. 2026 May 18;16(29):26432–7. doi: 10.1039/d6ra01273f (PMC13185705; doi:10.1039/d6ra01273f)
Supplement: RA-016-D6RA01273F-s001 [file RA-016-D6RA01273F-s001.pdf]

Supplementary Information (SI) for RSC Advances.
This journal is © The Royal Society of Chemistry 2026

Supplementary Material

Quantum Confinement Dominates Band Gaps, while Defects Lead the Photoluminescence in Silicon Nanowires

Aarti Diwan[1], Tharun J [1], Chandeep EC[1], Anand Mohan Shrivastav[1], Tulika Srivastava[2*], Rajesh Kumar[3] and Shailendra K. Saxena[1*]

[1]Optics and Nanoelectronics Laboratory (ONE), Department of Physics and Nanotechnology, College of Engineering and Technology, SRM Institute of Science and Technology, Kattankulathur, Tamil Nadu, India, 603203

[2]Department of Electronics & Communication, College of Engineering and Technology, SRM Institute of Science and Technology, Kattankulathur, Chennai 603203, India

[3]Materials and Device Laboratory, Department of Physics, Indian Institute of Technology Indore, Simrol, India 453552

*Corresponding author(s) e-mail: tulikas@srmist.edu.in, shailens@srmist.edu.in

1. SEM image of silicon nanowires (SiNWs) in cross sectional view

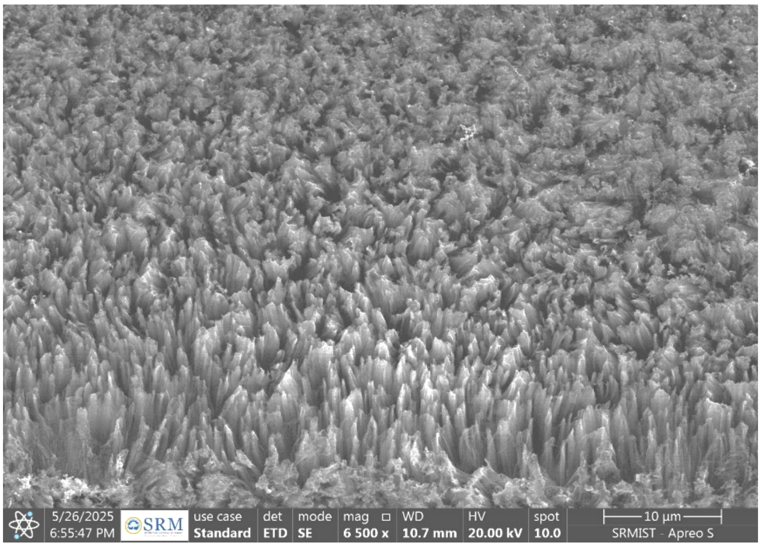

Figure 1. SEM image of cross- sectional view of silicon nanowires (SiNWs) for 30 minutes etching.

2. Examples of higher Urbach energies.

Material	Urbach energy (E_u)	References
Zinc oxide (ZnO	400 to 1000 meV	[1]
Tin oxide (SnO_2)	1,666 meV, 945 meV, and 12,210 meV	[2]
Erbium oxide ($Er2O_3$)	0.722 to 1.083 eV	[3]
Copper-doped hematite (Cu-Fe_2O_3)	1100 meV	[4]
mercuric-sodium-lead-borate glasses $(55\text{-}x)B_2O_3 \cdot 10SrF_2 \cdot 25PbO \cdot 10Na_2O \cdot xHgO$	0.74 to 1.54 eV	[5]
Poly (3, 4-ethylenendioxythiophene)	2197 to 2262 meV	[6]
Cadmium oxide (CdO)	715 to 826 meV	[7]
Phenylene thiophene based polyazomethines (PTPI)	1670 meV	[8]

References

1 D. Scolfaro, Y. J. Onofre, M. D. Teodoro and M. P. F. de Godoy, *International Journal of Photoenergy*, 2018, **2018**, 8607247.

2 F. Omoniyi, I. Omoteji and T. Imalero, 2026, **17**, 424–434.
3 A. S. Abouhaswa, U. Perişanoğlu, S. Saltık, N. Ekinci, M. H. Nasr, S. Kalecik and E. K. Perişanoğlu, *J Inorg Organomet Polym*, 2025, **35**, 4865–4883.
4 A. Y. Fasasi, E. Ajenifuja, E. Osagie, L. Animashaun, A. Adeoye and E. Obiajunwa, *Journal of the Nigerian Society of Physical Sciences*, 2023, 1180–1180.
5 Y. S. Rammah, A. T. Shah, O. Görke, N. V. Kudrevatykh and A. S. Abouhaswa, *Materials Research Bulletin*, 2023, **160**, 112136.
6 R. Ahmed, *Arab Journal of Nuclear Sciences and Applications*, DOI:10.21608/ajnsa.2018.2326.1024.
7 K. Haneen and D. Latif, *International Journal of ChemTech Research*, 2016, **9**, 332–338.
8 B. Jarząbek, B. Hajduk, M. Domański, B. Kaczmarczyk, P. Nitschke and H. Bednarski, *High Performance Polymers*, 2018, **30**, 1219–1228.
